# Supplementary material for: Content-rich biological network constructed by mining PubMed abstracts
Source: BMC Bioinformatics. 2004 Oct 8;5:147. doi: 10.1186/1471-2105-5-147 (PMC528731; doi:10.1186/1471-2105-5-147)
Supplement: Additional File 5 — The original Chilibot query results of the term "long-term potentiation (LTP)" and 22 other terms, limiting the latest references analyzed to the years 1990, 1995, 2000, and 2004. [file 1471-2105-5-147-S5.bz2 › chilibotAdditionalFile5/ltp2004/html/TRKA_CREB.html]

 


 **TRKA** and **CREB** 
  
Found 6 abstracts in PubMed,  **6 abstracts were retrieved and analyzed**.  


---

 Search Google  |
 PDF files only 
|  EDU domain only 

---

**Interactive relationship** (e.g. stimulation, inhibition, etc)

- The tyrosine kinase activity of  **TrkA**  was required to maintain it in an autophosphorylated state upon its arrival in the cell body and for propagation of the signal to  **CREB**  within neuronal nuclei.  Ref: 9262478 Science, 1997

**Parallel relationship** (e.g. studied together, co-existance, homology, etc.)

- Moreover, simultaneous activation of the A R PKA  **CREB**  mediated and the phosphatidylinositol 3 kinase pathways caused neurite outgrowth that was not suppressed by a selective inhibitor of  **TrkA** , indicating that transactivation of  **TrkA**  was not involved.  Ref: 12114502 J Biol Chem, 2002
- Thus, each component of the mitogen activated protein kinase pathway is crucially involved in relaying the nerve growth factor signal in trans to the chromogranin A gene, in the following proposed sequence nerve growth factor >  **TrkA**  > Shc Grb2 Sos > Ras > Raf > MEK > mitogen activated protein kinase > ribosomal protein S6 serine kinase II >  **CREB**  cyclic AMP response element.  Ref: 10197763 Neuroscience, 1999
